# A glimpse of practice of neurosurgery in Africa: Challenges and opportunities

**DOI:** 10.1016/j.bas.2023.102720

**Published:** 2023-12-02

**Authors:** Inibehe Ime Okon, Fadele Kehinde Precious, Minaam Farooq, Ibukunolu Olufemi Ogundele, Don Eliseo Lucero-Prisno, Bipin Chaurasia

**Affiliations:** Department of Research, Medical Research Circle (MedReC), Bukavu, People’s Republic of Congo; College of Medicine, University of Nigeria, Nsukka, Enugu state, Nigeria; Department of Neurological Surgery, Weill Cornell Medicine, New York, Presbyterian Hospital, USA; Department of Surgery, Olabisi Onabanjo University Teaching Hospital, Sagamu, Ogun State, Nigeria; Department of Global Health and Development, London School of Hygiene and Tropical Medicine, London, United Kingdom; Department of Neurosurgery, Neurosurgery Clinic, Birgunj, Nepal

Dear Editor,

Africa confronts a significant global neurosurgery challenge, which is anticipated by a workforce density of African neurosurgeons that is a lot higher than recommended. It depicts that there exists a substantial imbalance in the distribution of the surgical workforce, resulting in significant geographic gaps in healthcare access across the continent. This challenge can be attributed to numerous factors, encompassing insufficient healthcare planning, limited resources, inadequate support for the local training of neurosurgeons, and unfavorable conditions that hinder neurosurgeons from practicing effectively within the region. These challenges often result in neurosurgeons trained in Europe or elsewhere choosing not to return to their home countries (El Khamlichi, 2001). African countries, as a collective, face a substantial deficiency in the advancement of the field of neurosurgery. Countries on the continent of Africa, like other low- and middle-income countries (LMICs), bear the heaviest burden of unmet neurosurgical healthcare needs, which underscores the need for a concentrated emphasis on neurosurgery. The continent contends with a heightened incidence of conditions like traumatic brain injuries, cerebrovascular diseases, epilepsy, and neuroinfections, all of which demand specialized surgical interventions. Neurosurgery assumes a pivotal role in mitigating the distress of patients affected by these ailments, rendering it an indispensable specialty in our healthcare landscape.

Another pivotal factor is the harsh reality that, in low-income countries, individuals afflicted with neurosurgical conditions often lack the financial resources to access medical care for themselves. As a result, they frequently present at very advanced disease stages, making treatment more complex and contributing to higher levels of morbidity and mortality (Corley et al., 2019). One of the causes of the increased burden on healthcare in Africa is the constrained availability of specialized medical services, especially in remote and underserved regions. Neurosurgical practice is significantly reliant on the accessibility of surgical equipment, particularly in low- and middle-income countries (LMICs) where resources are constrained. The absence of prompt access to imaging tools results in delays in surgical interventions and unfavorable patient outcomes (Servadei et al., 2018). Furthermore, in addition to expanding the neurosurgical workforce, substantial efforts are required to establish the necessary infrastructure that enables African-trained neurosurgeons to effectively operate and advance their careers in the field of neurosurgery. These obstacles are even more challenging due to the highly specialized nature of neurosurgery, which depends on a complex network of interconnected processes. This network includes neuroimaging, neuro-anesthesia, specialized operating room requirements, dedicated nursing care, and rehabilitation services. Furthermore, the scarcity of practicing neurosurgeons, the inability to incorporate neurosurgical training into existing surgical education programs because of a lack of equipment and resources for diagnostic imaging, and an insufficient surgical infrastructure that limits the capacity of the few available neurosurgeons to provide comprehensive care all exacerbate the challenges. The dearth of neurosurgical infrastructure is exacerbated by a lack of resources and a culture of poor maintenance. This combination further obstructs access to neurosurgical services (Aderinto et al., 2022).

The provision of neurosurgery necessitates a sufficient quantity of highly trained, well-equipped, and highly motivated neurosurgeons. Numerous African-trained neurosurgeons seek opportunities abroad, largely driven by the challenges mentioned above. This “brain drain” phenomenon exacerbates the already limited availability of specialized medical professionals within the continent. However, several other factors make this practice challenging in Africa, including the geographical isolation of many communities, bureaucratic obstacles, political instability, frequent socioeconomic fluctuations, and elevated levels of poverty and illiteracy within rapidly expanding populations (Sader et al., 2017). The shortage of funding for healthcare, primarily driven by the lack of governmental commitment to health financing, in conjunction with the elevated poverty rates in Africa, amplifies the deficiency in access to specialized post-operative care (Aderinto et al., 2022). In certain countries, millions of citizens are deprived of access to neurosurgical care, and this predicament arises from insufficient governmental advocacy for neurosurgery rather than solely economic constraints. The insufficiency of neurosurgeons, neurologists, and other essential medical personnel constitutes a significant hindrance to the provision of effective neurosurgical care. Training programs for these specialists are often lacking, and retaining them within the continent poses a considerable challenge. When compared to more developed regions, an acute scarcity of neurosurgeons in numerous African nations and a substantial disparity in neurosurgical proficiency are observed. The need of the hour is that medical students and residents of Africa must undergo comprehensive training via observership and fellowship programs in developed countries and acquire expertise in this field of neurosurgery. They must have the ultimate goal of returning to Africa and actively participating in the enhancement of neurosurgical capabilities within African healthcare systems. It would create a significant impact by bringing specialized neurosurgical care closer to those who need it the most (Agarwal et al., 2013; Lartigue et al., 2021; Deora et al., 2020; Javed et al., 2023). The practice of neurosurgery relies on continuous research and innovation. The lack of neurosurgical research significantly hinders the advancement of neurosurgery, a situation notably evident in countries across Southeast Asia and Africa (Lartigue et al., 2021; Deora et al., 2020; Javed et al., 2023; Chaurasia, 2023a, 2023b; Chaurasia et al., 2023). However, the restricted resources and infrastructure in Africa impede the development of new techniques and technologies, thus impeding progress in the field. There is a need to implement initiatives like establishing collaborative partnerships between institutions in high-income countries (HICs) and low- and middle-income countries (LMICs) (Chaurasia, 2023c; Farooq et al., 2023). It would incentivize researchers to conduct and publish research that enriches the global neurosurgery literature within LMICs, resulting in opportunities for capacity-building (Servadei et al., 2018).

The difficulties in the practice of neurosurgery in Africa are multifaceted and deeply ingrained. These challenges are not isolated; they are interconnected and amplify one another.

In summary, the practice of neurosurgery in Africa confronts substantial challenges arising from resource constraints, insufficient infrastructure, and cultural factors. Nevertheless, through collaborative endeavors involving governments, medical practitioners, and the global community, it is possible to tackle these issues. By bolstering investments in healthcare, education, and research, we can enhance access to neurosurgical care, alleviate the burden of neurological conditions, and pave the way for a brighter future for our continent.

## Declaration of competing interest

None.
